# MicroRNAs Associated with Chronic Kidney Disease in the General Population and High-Risk Subgroups—A Systematic Review

**DOI:** 10.3390/ijms24021792

**Published:** 2023-01-16

**Authors:** Dipuo D. Motshwari, Don M. Matshazi, Rajiv T. Erasmus, Andre P. Kengne, Tandi E. Matsha, Cindy George

**Affiliations:** 1 SAMRC/CPUT/Cardiometabolic Health Research Unit, Department of Biomedical Sciences, Faculty of Health and Wellness Science, Cape Peninsula University of Technology, Cape Town 7530, South Africa; 2 Division of Chemical Pathology, Faculty of Medicine and Health Sciences, National Health Laboratory Service (NHLS) and University of Stellenbosch, Cape Town 7505, South Africa; 3 Non-Communicable Disease Research Unit, South African Medical Research Council, Parow, Cape Town 7505, South Africa; 4 Department of Medicine, University of Cape Town, Cape Town 7925, South Africa; 5 Sefako Makgatho Health Sciences University, Ga-Rankuwa 0208, South Africa

**Keywords:** chronic kidney disease, diabetic kidney disease, hypertension-associated CKD, micro-RNAs

## Abstract

The potential utility of microRNAs (miRNAs) as diagnostic or prognostic biomarkers, as well as therapeutic targets, for chronic kidney disease (CKD) has been advocated. However, studies evaluating the expression profile of the same miRNA signatures in CKD report contradictory findings. This review aimed to characterize miRNAs associated with CKD and/or measures of kidney function and kidney damage in the general population, and also in high-risk subgroups, including people with hypertension (HTN), diabetes mellitus (DM) and human immunodeficiency virus (HIV) infection. Medline via PubMed, Scopus, Web of Science, and EBSCOhost databases were searched to identify relevant studies published in English or French languages on or before 30 September 2022. A total of 75 studies fulfilled the eligibility criteria: CKD (n = 18), diabetic kidney disease (DKD) (n = 51) and HTN-associated CKD (n = 6), with no study reporting on miRNA profiles in people with HIV-associated nephropathy. In individuals with CKD, miR-126 and miR-223 were consistently downregulated, whilst in DKD, miR-21 and miR-29b were consistently upregulated and miR-30e and let-7a were consistently downregulated in at least three studies. These findings suggest that these miRNAs may be involved in the pathogenesis of CKD and therefore invites further research to explore their clinical utility for CKD prevention and control.

## 1. Introduction

The incidence of chronic kidney disease (CKD) is on the rise globally, and it is expected that CKD will be the fifth leading cause of death by 2045 [1]. This is partly attributable to the high burden of diabetes mellitus (DM) and hypertension (HTN), which are the leading causes of CKD, as well as other causes, including human immunodeficiency virus (HIV) infection and advanced age [2]. Chronic kidney disease is described as a silent condition due to a lack of obvious clinical symptoms, particularly in its early stages. As a result, most affected individuals are unaware of their disease status, and are often only detected at an advanced stage of the disease [3]. Furthermore, CKD is an independent risk factor for cardiovascular disease (CVD), and individuals with CKD are more likely to die of CVDs than progress to end-stage kidney disease (ESKD) [4]. Early diagnosis of CKD and effective screening of high-risk individuals is critical to mitigate disease progression and substantially reduce related poor health outcomes [5].

The indirect measurement of glomerular filtration rate (GFR) by clearance of exogenous filtration markers remains the reference standard method for determining kidney function. However, this method is complex, lengthy, expensive, invasive, and as such, not ideal for routine practice or research purposes [6]. As a result, endogenous filtration markers such as serum creatinine and cystatin C are used to estimate GFR (eGFR), and kidney function in clinical practice. However, serum creatinine can be affected by factors independent of glomerular filtration such as muscle mass, age and gender, whereas the measurement of cystatin C is complex, expensive and has not been standardized [7]. Furthermore, the predictive equations for eGFR are biased and imprecise, translating into overestimation of GFR and underdiagnosis of CKD, particularly in black Africans [8,9]. Although, albuminuria is a well-established marker of kidney damage used to define stages 1 and 2 of CKD where the level of GFR is above 60 mL/min/1.73 m^2^, it has limited predictive ability and specificity for early detection of CKD [10]. Kidney biopsies can be used to confirm a diagnosis, but this option comes with significant risk and possibility for complications, and therefore is not ideal for routine practice or research purposes [11]. Put together, these diagnostic challenges highlight the need for more accurate, minimally invasive, highly sensitive and specific, and readily available biomarkers that will improve the diagnosis/prognosis of CKD.

Research into microRNAs (miRNAs) as potential biomarkers of disease diagnosis and prognosis, as well as therapeutic targets, has gained traction over the last 10 years [12]. MiRNAs are a class of small non-coding RNAs, whose main function is regulating gene expression by degrading messenger RNA (mRNA) or inhibiting mRNA translation into functional proteins [13]. They play an important role in various cellular regulatory processes, such as differentiation, proliferation, development and apoptosis [13], and are also involved in the development and normal functioning of the kidneys [14]. Although they were initially considered to be intracellular gene regulators, emerging evidence suggests that a number of miRNAs are also detectable in biological fluids, such as urine, plasma, serum, and saliva in highly stable forms [15]. Previous studies found that these extracellular miRNAs presented unique patterns in pathological conditions and suggested that they may be utilized as potential diagnostic and prognostic biomarkers [16,17,18,19]. There has been a growing interest in exploring the role of extracellular miRNAs in the development and progression of CKD [19,20,21,22]. However, most findings describing miRNA expression in various biological fluids from CKD patients are inconsistent.

As such, the main purposes of this review were: (1) to identify all reported miRNAs associated with CKD and/or measures of kidney function and kidney damage in the general population, as well as in high-risk subgroups (HTN, DM and HIV-infected), and (2) to explore the specific expression patterns of the identified miRNAs in prevalent CKD. We also aimed to explore (3) whether the expression patterns of the identified miRNAs differed depending on the human sample type used and/or 4) whether the expression profile of the identified miRNAs differed depending on the stage of CKD.

## 2. Materials and Methods

### 2.1. Protocol and Registration

This review was conducted in accordance with the Preferred Reporting Items for Systematic Reviews and Meta-Analyses (PRISMA) guidelines. The protocol for this systematic review was registered on the PROSPERO database (Registration No. CRD42021270028), and detailed methods outlining the steps followed in conducting the systematic review have been previously published [23].

### 2.2. Search Strategy

A comprehensive and systematic search of Medline via PubMed, Scopus, Web of Science, and EBSCOhost databases was conducted to identify eligible studies, published in English or French languages on or before 30 September 2022, without a starting date. The search strategy made use of keywords and phrases such as “microRNAs, miRNA, miRNAs, chronic kidney disease, CKD, chronic kidney injury, chronic renal disease, chronic renal injury, renal failure, end-stage renal disease, diabetic kidney disease, diabetic nephropathy, hypertensive nephrosclerosis, chronic kidney failure, chronic renal failure, end-stage renal failure, HIV-associated nephropathy, HIVAN, HIV-associated renal disease, HIV-associated kidney disease, serum creatinine, serum cystatin C, estimated glomerular filtration rate, urinary albumin excretion rate (UAER), urinary albumin-to-creatinine ratio (UACR), urinary albumin” in combination with Boolean operators (AND/OR/NOT) (refer to Additional files: Tables S1–S4). Furthermore, we manually scanned reference lists of the included studies for additional studies.

### 2.3. Data Collection

**Figure 1:**
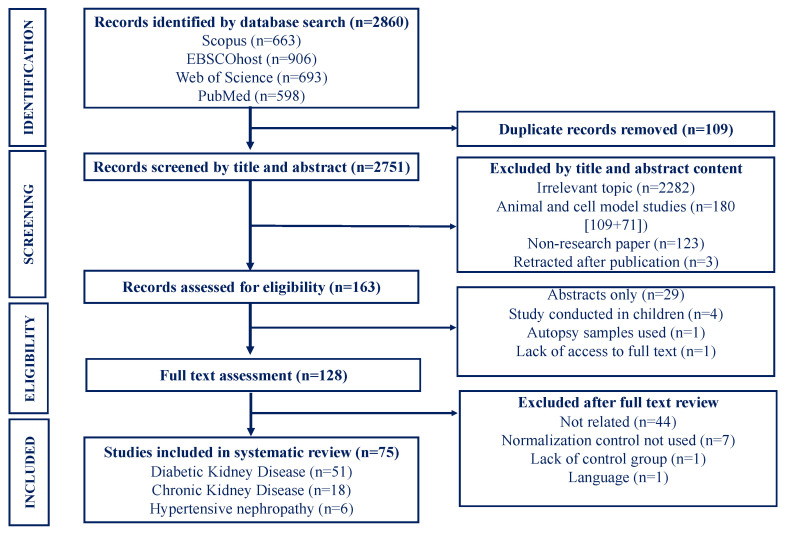
Selection process for studies included.

Two authors (DDM and DMM) independently conducted the database searches and screened studies by title, abstract and full text to identify those meeting the inclusion criteria, as shown in Figure 1. Disagreements encountered were resolved through discussions or consultation with a third author (CG). Studies were included if they: (i) were original articles reporting on miRNAs associated with prevalent CKD and/or measures of kidney function (serum creatinine, serum cystatin C, eGFR) or kidney damage (urinary albumin excretion rate, albumin-to-creatinine ratio, urinary protein) in the general adult population and/or high-risk subgroups (HTN, DM, HIV-infection), ii) written in English and French languages, (iii) clearly described the type of sample in which miRNA analysis was done, methods used for miRNA detection and quantification, as well as the normalization control used, (iv) with clearly defined cases and controls. Studies were excluded if they were: (i) conducted in animal or cell models, (ii) qualitative in nature (reviews, case reports, newspaper articles, editorials, commentaries, book chapters), or (iii) pre-prints or unpublished research.

### 2.4. Data Extraction, Assessment, and Synthesis

The following data were independently extracted by two reviewers (DDM and DMM) from the eligible studies: publication details (first author, year of publication, country); study details [design, sample size, demographics (age, sex)]; disease outcome [CKD (of unspecified cause), diabetic kidney disease (DKD)/HTN-associated CKD/HIV-associated nephropathy (HIVAN) or ESKD]; population [general or high-risk subgroups (HTN, DM and HIV)]; participant clinical characteristics [body mass index (BMI), C-reactive protein (CRP), smoking status, alcohol consumption, lipid profile (low-density lipoprotein (LDL), high-density lipoprotein (HDL), triglycerides and total cholesterol)]; clinical outcomes (diagnostic criteria, classification/staging, medication status); CKD diagnostic criteria (eGFR or proteinuria/albuminuria and eGFR equation used); miRNA analysis [sample type, molecular techniques, inclusion of screening and validation cohorts, expression pattern (upregulated or downregulated) and normalization control used]. Any inconsistencies or disagreements were resolved by discussions or consultation with a third author (CG). Furthermore, we assessed the quality of studies using the Newcastle-Ottawa Quality Assessment Scale for observational studies (NOS) tool [24]. The assessment was done based on a critical appraisal of three domains, namely: (i) participant group selection, (ii) how comparable the groups are, and (iii) determination of the exposure of interest. The quality of evidence was then assessed using the Grading of Recommendations Assessment, Development and Evaluation (GRADE) framework [25]. Given that very few studies investigated the association of the same miRNA with CKD risk or markers of kidney function or damage in the same sample type, making use of different normalization controls and miRNA quantification techniques as well as variabilities in disease outcome measures, and attempting to pool studies was meaningless. We, therefore, instead opted for a narrative synthesis of evidence.

## 3. Results

### 3.1. Search Results

We obtained a total of 2860 related citations (663 from Scopus, 906 from EBSCOhost, 693 from Web of Science and 598 from PubMed) from database searches. Of these, 2732 citations were excluded for various reasons (Figure 1). The remaining 128 articles were further assessed for eligibility and 53 studies were subsequently excluded from the review because they were irrelevant to our review (n = 44); they did not report on the quantitative reverse transcription polymerase chain reaction (RT-qPCR) normalization controls used (n = 7), did not include a control group (n = 1) and did not meet our reporting language restrictions (n = 1). Ultimately, 75 studies fulfilled the eligibility criteria and were retained for the systematic review (Figure 1). The eligible studies were classified according to disease outcome, as follows: CKD (n = 18), DKD (n = 51) and HTN-associated CKD (6). Database searches did not return studies reporting on miRNA profiles in humans with HIVAN.

### 3.2. Characteristics of Included Studies

Table 1, Table 2 and Table 3 detail the main characteristics of studies that were included in the systematic review according to disease outcome. Table 1 summarizes the 18 studies that quantified miRNA expression patterns in CKD compared to controls, whilst Table 2 is a summary of the 51 studies that quantified miRNA expression patterns in DKD relative to controls. A summary of the six studies that quantified miRNA expression patterns in individuals with HTN-associated CKD compared to controls is shown in Table 3. All 75 studies were published between 2013 and 2022, and from diverse geographical locations, including China (n = 26), the United States of America (n = 6), Spain (n = 4), Egypt (n = 6), South Africa (n = 1), Germany (n = 3), Italy (n = 2), Austria (n = 2), Japan (n = 3), Iran (n = 3), Belgium (n = 1), Sweden (n = 1), Turkey (n = 1), Poland (n = 1), Bahrain (n = 2), Brazil (n = 2), United Kingdom (n = 1), India (n = 2), Romania (n = 2), France (n = 1), Canada (n = 1), Ireland (n = 1), Republic of Korea (n = 1), Netherlands (n = 1) and Malaysia (n = 1). The design of most studies was either case-control or cross-sectional, with study participant numbers ranging between 28 to 1385 in CKD, 11 to 1018 in DKD and 30 to 150 in HTN-associated CKD. The included studies used varying diagnostic methods to classify kidney disease, with 20% of the studies using only eGFR to classify CKD, 42% defined CKD by the level of albuminuria alone, whilst 34% of the studies used both albuminuria and eGFR to classify CKD. The remaining 4% of studies were not clear on the methods used for CKD classification. For estimation of GFR, most studies used the Modification of Diet in Renal Disease Study (MDRD) equation [26] (44% of the studies) and Chronic Kidney Disease Epidemiology Collaboration (CKD-EPI) equation [27] (38% of the studies). Of those included, 26% of the studies further validated their findings in a separate cohort [CKD (n = 3), DKD (n = 14) and HTN with CKD (n = 1)].

Of the included studies, 29% performed initial miRNA expression discovery using next-generation sequencing (NGS) techniques [CKD (n = 2); DKD (n = 2), HTN with CKD (n = 4)] and microarrays [CKD (n = 1); DKD (n = 15)], followed by a validation step using a PCR-based technique. PCR-based techniques were used for the quantification of miRNAs in instances where the miRNA was already identified by NGS and microarrays in previous studies. A wide range of normalization techniques were employed by the studies included in this review, with 31% using an exogenous spike-in control containing non-mammalian synthetic miRNAs such as *Caenorhabditis elegans*-miR-39 (cel-miR-39) (n = 22) and 52% of the studies used endogenous controls such as small non-human ubiquitous miRNA (U6) (n = 28) or miR-16 (n = 8). Of the included studies, 15% of the studies used more than one normalization control [CKD (n = 4), DKD (n = 5) and in HTN-associated CKD (n = 2)]. The sample types in which miRNA expression levels were determined varied widely across studies, with 36% conducted in serum [CKD (n = 13) and DKD (n = 17)], 21% in plasma [CKD (n = 2), DKD (n = 10) and HTN-associated CKD (n = 4)], 1% in plasma endothelial vesicle [DKD (n = 1)], 8% in whole blood [CKD (n = 1), DKD (n = 4), and HTN-associated CKD (n = 1)], 19% in urine [CKD (n = 2), and DKD (n = 12)], 19% in urinary exosomes [CKD (n = 2), DKD (n = 8) and HTN-associated CKD (n = 2)], 7% in kidney tissue biopsy [CKD (n = 1) and DKD (n = 4)] and 1% peripheral blood mononuclear cells [DKD (n = 1)]. Only 17% of the studies quantified miRNA expression in two or more sample types, and this included two studies in CKD, 10 in DKD and one in HTN-associated CKD.

The expression patterns of 288 miRNAs were investigated across the 75 studies included in this review. Of the 288 miRNAs, 67 miRNAs were evaluated in populations with prevalent CKD, with 53 miRNAs found to be dysregulated (25 downregulated and 28 upregulated). Of the 193 miRNAs evaluated in populations with DKD, 155 miRNAs were found to be dysregulated (67 downregulated and 88 upregulated), whilst 13 (10 downregulated and 3 upregulated) of the 28 miRNAs evaluated in populations with HTN-associated CKD were dysregulated. The dysregulation discussed below refers only to miRNAs evaluated in three or more studies, of which HTN-associated CKD had none.

### 3.3. Dysregulated miRNAs in CKD

Of the 53 differentially expressed miRNAs in individuals with CKD, four miRNAs (miR-126, miR-223, miR-155 and miR-21) were reported in at least three studies (Table 4). Of these, serum miR-126 [21,28,32] and serum miR-223 [21,28,32,43] were consistently downregulated in individuals with CKD across studies. Inconsistent expression patterns were observed for miR-155, two studies found the miRNA to be downregulated in serum and urine, [29,30] and two studies found it upregulated in serum [30,31] in CKD, with one study reporting no difference [41] in the expression of miR-155 in both urine and serum, in individuals with and without CKD. Of the studies reporting on the expression pattern of miR-21 in CKD, one study showed a downregulation in this miRNA in serum sample [30], whilst two studies evaluated miR-21 in urine and urine exosomes and found it upregulated [30,34], and one study found no difference in urine and plasma samples between the case and control groups [40].

### 3.4. Dysregulated miRNAs in DKD

One hundred and ninety-three miRNAs were differentially expressed in DKD, and of these, 12 miRNAs (miR-155, miR-126, miR-192, miR-21, miR-29b, miR-15a-5p, miR-29a, miR-29c, miR-124, let-7a, miR-30e and miR-30b) were reported in at least three studies. miR-21 [51,60,61,64,67,71,76,77,89] and miR-29b [50,53], were consistently upregulated whereas let-7a [19,74,93] and miR-30e [19,57,74] were consistently downregulated in individuals with DKD across studies (Table 5). Although discordant results were observed for miR-155, miR-126 and miR-192, they were commonly studied in at least four different studies. miR-155 was downregulated in DKD in three different studies [19,47,52], with one study reporting upregulation [53]. In the five studies where miR-126 expression was investigated in serum, three different studies showed consistent upregulation of the miRNA [53,60,64], whilst two studies reported downregulation [48,76], in individuals with DKD and controls. Three studies reported downregulation of miR-192 [49,70,71], whilst three other studies [64,66,76] observed upregulation of this miRNA in individuals with DKD compared to controls.

### 3.5. MicroRNAs Associated with Kidney Disease Subgroups

**Figure 2:**
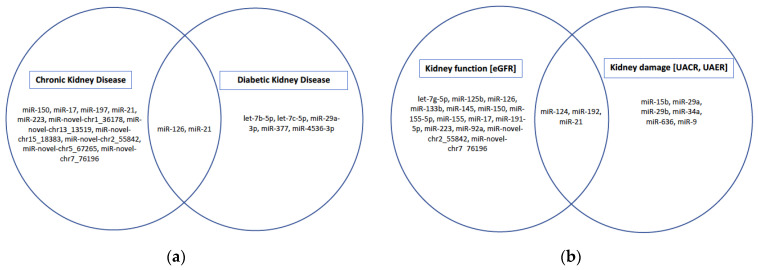
Associations between miRNAs and CKD subgroups and markers of kidney function and damage. (**a**) Shows miRNAs independently associated with CKD in general population and in high-risk individuals with HTN and DM. (**b**) miRNAs associated with markers of kidney function and or kidney damage in general population and in high-risk individuals with HTN and DM.

Studies reporting on the independent associations of various miRNAs with kidney disease outcomes and/or markers of kidney function and kidney damage are summarized in Table 5 and Table 6, as well as in Figure 2. In the general population, miR-17 [22], miR-21 [22], miR-150 [22], miR-197 [32] and miR-223 [32] were inversely associated with prevalent CKD, whilst miR-novel-chr2_55842, miR-novel-chr7_76196, miR-novel-chr5_67265, miR-novel-chr13_13519, miR-novel-chr1_36178 and miR-novel-chr15_18383 [37] were positively associated with prevalent CKD. In individuals with DM, miR-377 [49] was inversely associated with DKD, whereas miR-4536-3p [54] was positively associated with DKD. MicroRNAs, let-7b-5p, miR-21-5p were positively associated with progression to ESKD, whereas let-7c-5p, and miR-29a-3p were inversely associated with progression to ESKD in those with DM [77]. In the general population, miR-126 was inversely associated with the risk of CKD [32,33], whereas in individuals with DM it was inversely associated with DKD, microalbuminuria and macroalbuminuria [48]. In individuals with HTN-associated CKD, miR-29a [95] and miR-29b [96] were positively associated with albuminuria.

## 4. Discussion

Recent studies have demonstrated that miRNAs are key mediators in the pathophysiology of CKD, suggesting that circulating miRNAs have potential utility as alternative markers for early detection and progression of CKD, as well as monitoring treatment responses. Circulating and urinary miRNAs are ideal minimally- or non-invasive biomarkers because they are stable in body fluids and exosomes and can be detected using validated techniques for quantification. However, miRNA profile studies in humans have shown contradictory results, with few miRNAs being consistently dysregulated across studies. We performed a systematic review of studies that evaluated miRNA expressions in CKD in the general population, and high-risk subgroups (individuals with HTN and DM). We found that two miRNAs (miR-126 and miR-223) were consistently downregulated in the general population with CKD, whilst miR-21 and miR-29b were consistently upregulated and let-7a-3p and miR-30e were consistently downregulated in individuals with DKD, in whole blood, plasma, serum, urine, or urinary exosomes. Although showing inconsistent data, miR-155, miR-192, miR-15a-5p, miR-29a, miR-29c were also commonly quantified in the studies included in this review. Of note, only a few studies quantified miRNA expression in individuals with HTN-associated CKD, and reported inconsistent findings and none in HIVAN.

MiR-126 is endothelial cell-specific and promotes vascular integrity and angiogenesis via regulation of vascular endothelial growth factor (VEGF) signalling and, as a result, inhibits vascular inflammation [100]. MiR-126, which was downregulated in CKD in the general population [21,28,32] and individuals with DM [48,76] and HTN [99] when quantified in serum and whole blood samples, was inversely associated with prevalent CKD [32,33] and positively associated with eGFR [21,33]. A prospective study showed that lower levels of miR-126 were associated with an increased risk of developing CKD and rapid decline in kidney function over a period of five years [33]. Zhou et al. also demonstrated that miR-126 has an atheroprotective role, as it increases vascular smooth muscle cells (VSMCs) turnover, thereby regulating the contractile phenotype of VSMC [101]. Taken together, the downregulation of miR-126 may result in vascular dysfunction, which is very common in early-stage CKD. MiR-126 may therefore be a potential biomarker for the early identification of CKD and a potential target for the prevention or treatment of CKD-related vascular complications. Contrary to these findings, a few studies reported upregulated expression of miR-126 individuals with DKD [53,60,64,76] when quantified in urine, plasma as well as serum. It is plausible that this may be a compensatory mechanism resulting in increased release of miR-126 when microvascular endothelial cells are exposed to stressful conditions [102]. Indeed, Beltrami and colleagues used in vitro analyses and showed that miR-126 is released from glomerular endothelial cells in response to DKD-related cytokines [53].

Similarly to miR-126, miR-223 also plays a role in the regulation of VSMC proliferation [103]. This miRNA was consistently downregulated in the general population with CKD in serum samples [21,28,32,43]. Moreover, lower levels of this miRNA were associated with lower levels of eGFR [32], and prevalent CKD [21]. Although commonly considered to be involved in inflammatory pathways, evidence also suggests a protective role of miR-223 in VSMCs, through the inhibition of calcification by the regulation of interleukin-6 (IL-6)/signal transducer and activator of transcription 3 (STAT3) [63]. These findings imply that increased levels of miR-223 may improve kidney function, and thus may serve as a therapeutic strategy to improve CKD outcomes in the general population.

The involvement of miR-21 in kidney fibrosis is well established, although the mechanisms involved have not been completely clarified. miR-21 acts as a pro-fibrotic factor, and its upregulation induces kidney fibrosis through TGF-β signalling pathway regulation [104]. Consistent upregulation of miR-21 in individuals with DKD [51,61,64,67,71,76,77,89] as well as its inverse association with eGFR [71] and positive association with albuminuria [71] have been reported. Moreover, increased levels of miR-21 were associated with rapid progression to ESKD over a 10-year follow-up period [77]. Correspondingly, in vitro and in vivo knockdown of miR-21 ameliorated DKD by reducing albuminuria, kidney inflammation, podocyte loss and interstitial fibrosis, suggesting its value as a potential therapeutic target against DKD progression [105]. Although the observed findings imply that increased expression of miR-21 may be associated with the development and progression of DKD, the data were inconclusive in the case of CKD. In the general population with CKD, contrasting results were reported, with miR-21 upregulated in urine [30] and urine exosomes [34] but downregulated in serum samples [30]. Moreover, Fujii and colleagues found that increased levels of miR-21 were positively associated with eGFR and inversely associated with the risk of CKD in the general population [22]. Donderski et al. (2021) explained that the lower levels of miR-21 detected in serum samples of individuals with CKD may be as a result of suppression caused by increased fibrosis and TGF–β activity [30]. This is in line with the findings by Sun et al. (2018), using a murine kidney fibrosis model, they observed that miR-21 is the main regulator of fibroblasts activation through an auto-regulatory loop between miR-21 and programmed cell death protein 4 and activated protein 1, therefore suggesting that miR-21 may act as pro- or anti-fibrotic depending on the cell type [106]. It has been suggested that identification of the cellular source of miRNAs would be helpful instead of the biofluid sample to link the miRNAs to the specific disease process [107].

The miR-29 family has been well studied with regard to the TGF-β signalling pathway [104]. Two studies included in our review reported that miR-29b was upregulated in the urine samples of individuals with DKD [50,53], although one study reported no difference in the expression level of miR-29b when quantified in urine supernatant [75]. The lack of difference could be explained by the relatively lower abundance of miR-29b in urine supernatant sample reported in this study [75]. In individuals with HTN, increased expression of miR-29b was positively associated with albuminuria and inversely associated with kidney function [96]. However, these findings are contrary to previous studies that have reported on the protective role of this miRNA in DKD. Chen et al. (2014) showed that knockdown of miR-29b in diabetic mice was associated with increased albuminuria and TGFβ mediated fibrosis whereas overexpression of miR-29b attenuated kidney fibrosis in DKD through the regulation of TGFβ1/Smad3 pathway [108]. The upregulated expression of miR-29b in DKD observed in the included studies in our review may be due to the compensatory release of miR-29b. Beltrami and colleagues used in vitro analyses and observed increased release of miR-29b from glomerular endothelial cells in response to DKD-related cytokines [53]. Regarding the expression profile of miR-29a and miR-29c, contradictory results were observed when quantified in various samples of individuals with DKD. miR-29a was downregulated in plasma samples of individuals with severe DKD [51] and inversely associated with rapid progression to ESKD over a 10-year follow-up period [77], suggesting that this miRNA may have a protective effect against the progression of DKD. However, when quantified in urine supernatant, upregulated expression of miR-29a was observed in individuals with DKD [75]. Studies have previously highlighted that urine supernatant miRNAs inversely reflect intracellular miRNAs [109], which could be the possible reason for the discrepancy, however, tissue expression of miR-29a was not analyzed in this study [75]. On the other hand, Guo et al. (2017) [62] analyzed the expression of miR-29c in three different samples of individuals with DKD relative to those without, and found downregulated expression in urinary sediments and kidney tissues but upregulated expression in plasma. Cui and Cui (2020) found that relative to blood, urinary miRNAs highly reflected kidney tissue miRNAs and suggested that urine should be a better sample for kidney miRNAs analysis [110].

MicroRNAs miR-30e and let-7a were consistently downregulated in individuals with DKD relative to those without DKD, suggesting that increased expression of these miRNAs may have protective effects in the kidney and therefore may serve as possible diagnostic and prognostic markers of DKD. Accordingly, previous evidence suggests that the let-7 family of miRNAs may be a negative regulator of kidney fibrosis in DKD [111]. Muralidharan and colleagues [38] validated the expression of let-7a in an Alb/TGFβ mouse model of CKD and found that the miRNA was significantly downregulated further suggesting its possible role in the development or progression of CKD. MicroRNAs in the miR-30 family are highly enriched in kidney podocytes cells where they are involved in regulatory roles and are essential for structural and functional homeostasis [112]. In vitro and in vivo experimental studies showed that the expression of miR-30e was significantly decreased in those with DKD whereas overexpression of miR-30e was protective against the development of kidney fibrosis in DKD suggestive the potential role of this miRNA as a therapeutic target [113].

The miR-155 was commonly analyzed in the studies included in this review. Experimental studies have shown that suppressing miR-155 expression in DKD mice protects against kidney damage, attenuates hyperglycaemia-induced kidney damage and downregulates IL-17 expression by enhancing the suppression of cytokine signalling 1 (SOCS1) [114]. In line with these, upregulated expression of miR-155 was observed in the general population with CKD [30,31] and individuals with DKD 53. However, downregulated expression of miR-155 was commonly reported in individuals with DKD [19,47,52], as well as in the general population with CKD [29,30]. Furthermore, Donderski and colleagues reported on the positive association of miR-155 and eGFR [30]. These findings suggest that miR-155 may also play a role in the development of DKD. Wang et al. (2018) demonstrated that miR-155 is involved in the regulation of the autophagic process in DKD through the regulation of a signalling loop p53/miR-155-5p/Sirt1 and may therefore serve as a therapeutic strategy for DKD [115].

miR-192 has been shown to have a protective effect against kidney fibrosis. Downregulated expression of miR-192 was observed in individuals with DKD [49,70,71], and reduced levels of miR-192 were positively associated with kidney function [71] and inversely associated with kidney damage [70,71], suggesting that miR-192 levels may decrease with the increasing level of albuminuria and a decline in kidney function. Consistently, in vivo studies have shown that loss of miR-192 was associated with the development and progression of DKD through exacerbation of kidney fibrosis by enhancing TGF-β1 signalling pathway [116]. These findings suggest that reduced expression of miR-192 in the early stage may be associated with the development of DKD and therefore may serve as an early indicator of DKD. However, contrary to these findings, increased expression of miR-192 in individuals with DKD relative to controls has been observed in a few studies [64,76]. Jia and colleagues reported that the expression of miR-192 was increased during the early stages of DKD and decreased in the advanced stages of DKD [66]. They further showed that miR-192 was positively correlated with albuminuria and TGF-β1 levels [66], suggesting a profibrotic role of this miRNA. Jenkins et al. (2012) highlighted that miR-192 is pleiotropic, involved in multiple important roles in the kidney, and its role as an antifibrotic or profibrotic factor may be cell dependent [117].

This review provides an overview of miRNA dysregulation in CKD, including diabetic and hypertensive-related CKD in humans. It also highlights miRNAs that are associated with CKD and its clinical indicators. A few miRNAs showed consistent expression patterns in CKD relative to controls, whilst most of the frequently studied miRNAs showed contradictory findings. These discrepancies may be partly explained by the technical and methodological inter-study variabilities, such as the use of different biological samples, sample handling and processing procedures, miRNA extraction protocols, detection and quantification techniques, and normalizing controls [118]. Although the majority of included studies quantified miRNA expression in blood, recent evidence points to the superiority of urine miRNAs to serum/plasma miRNAs for CKD diagnosis with the non-invasive nature in which urine samples are collected, adding to its preference [119]. Another challenge is the lack of a standardized normalization control for miRNA expression studies. Although a wide range of endogenous and exogenous miRNAs are employed as normalizers, emerging evidence suggests that the use of synthetic spike in controls such as cel-miR-39 is preferable [120]. Therefore, to be able to identify reliable miRNA biomarkers, research findings need to be reproducible and comparable between studies. This can be achieved when normalization controls have been validated, and there is a standardization of robust protocols for sample processing and extraction [121].

### Strength and Limitations

The main strength of this review is its comprehensive report of miRNAs dysregulated in CKD, their association with CKD as well as clinical markers of CKD in the general population as well as in high-risk individuals with HTN and/or DM for the very first time. The review also provides a list of miRNAs that have been frequently studied in diverse geographical areas and showed consistent expression patterns across studies in CKD and DKD and therefore are worthy of future research.

The studies included in the review had their own shortcomings and, as such, impacted the review’s overall quality of evidence. The most important limitation of the review was the inability to perform a pooled meta-analysis of our studies due to various factors, including insufficient raw data on fold changes or relative expression of miRNAs, technical and methodological variabilities between studies, such as the use of different biological samples, normalization control used, and different miRNA quantification techniques. Moreover, due to insufficient data, we could not report on the expression patterns of miRNAs across different stages of CKD. The language restriction on the inclusion criteria may have excluded other relevant studies, thus biasing our findings. Additionally, there were differences in the classification of CKD, wide sample size ranges, variability in participant demographic factors such as age, sex proportion, and race, as well as environmental and regional factors between studies.

## 5. Conclusions

MiRNAs detected in biofluids are promising as potential biomarkers of disease diagnosis and therapeutic targets for future clinical applications. However, understanding their role in CKD pathophysiology and how their expression pattern is regulated is still in its infancy. As such, further research is required to fully elucidate their roles before any extrapolation for clinical use. Prevention and early detection of CKD has been a topic of interest for many researchers and clinicians in the field. This review highlights several dysregulated miRNAs that were frequently studied and showed consistent findings across studies in CKD (miR-126 and miR-223) or DKD (miR-21, miR-29b, let-7a and miR-30e) with a potential for clinical application in CKD diagnosis/prognosis in the future. This consistent alteration of miRNAs with CKD/DKD and their stability and detectability in bodily fluids suggests that these miRNAs are promising potential non-invasive or minimally invasive diagnostic markers for early detection and therapeutic targets of CKD/DKD and warrant further scrutiny in future investigations. Besides these specific miRNAs, miR-155, miR-192, miR-15a-5p, miR-29a and miR-29c, despite their inconsistent expression patterns reported in different studies, were commonly dysregulated in CKD and/or DKD, and therefore may also play an important role in CKD. As such, their further exploration is warranted. Furthermore, it may be worthwhile for future studies to focus on identifying target genes and pathways of these frequently studied miRNAs, to get a complete understanding of their role in the development and progression of CKD, as well as to assess their potential value as diagnostic markers or therapeutic targets. 

## Figures and Tables

**Table 1:** Characteristics of studies evaluating microRNA expression patterns in chronic kidney disease.

Study	Country	Study Population [Cases]	Study Population [Control]	Quantification Method	Sample Type	microRNAs	Upregulated	Downregulated
Carmona, 2020 [28]	Spain	45	10	RT-qPCR	Serum	miR-126-3p, miR-191-5p, miR-223-3p, miR-363-3p, miR-495-3p	-	miR-126-3p, miR-191-5p, miR-223-3p
Chen, 2013 [29]	United States of America	110	8	RT-qPCR	Serum	miR-125b, miR-145, miR-155	-	miR-125b, miR-145, miR-155
Donderski, 2021 [30]	Poland	45	17	RT-qPCR	urine, serum	miR-155-5p, miR-214-3p, miR-200a-5p, miR-29a-5p, miR-21-5p, miR-93-5p, miR-196a-5p	Urine—miR-29-5p, miR-21-5p, miR-196a-5p. Serum—miR-155-5p, miR-214-3p and miR-200a-5p	Urine—miR-155-5p, miR-214-5p, miR-200a-5p, miR-93-5p
Eckersten, 2017 [31]	Sweden	30	18	RT-qPCR	Serum	miR-155	miR-155	-
Fourdinier, 2019 [21]	Belgium	601	31	RT-qPCR	Serum	miR-223, miR-126	-	miR-223, miR-126
Fujii, 2019 [22]	Japan	395	118	RT-qPCR	Serum	miR-17, miR-21, miR-150	-	-
Fujii, 2019 [32]	Japan	229	1156	RT-qPCR	Serum	miR-126, miR-197, miR-223	-	miR-126, miR-197, miR-223
Fujii, 2021 [33]	Japan	29	140	RT-qPCR	Serum	miR-126, miR-197, miR-21, miR-150, miR-17	-	-
Lange, 2019 [34]	Germany	41	5	RT-qPCR	urine exosomes	miR-21-5p, miR-30a-5p, miR-92a-3p	miR-21	-
Li, 2020 [35]	China	116	127	RT-qPCR	Serum	miR-155	miR-155	-
Liu, 2020 [36]	China	110	35	NGS, RT-qPCR	serum	miR-483-5p, miR-363-3p	miR-483-5p	miR-363-3p
Motshwari, 2021 [37]	South Africa	171	740	NGS, RT-qPCR	whole blood	miR-novel- chr1_36178, miR-novel-chr2_55842, miR-novel-chr7_76196, miR-novel-chr5_67265, miR-novel-chr13_13519, and miR-novel-chr15_18383	All novel miRNAs	-
Muralidharan, 2017 [38]	United States of America	19	9	Microarray, RT-qPCR	plasma and urine exosomes	Urine—miR-1281, miR-1825, miR-130a-3p, let-7a-5p Plasma—miR-1825p miR-1281, miR-423	Urine—miR-1825, miR-1281. Plasma—miR-1825, miR-1281, miR-144-5p, miR-548ap-5p	Urine—miR-4525. Plasma—miR-423-5p, miR-3648
Rudnicki, 2016 [39]	Austria	20	52	RT-qPCR	Kidney biopsy	miR-30d, miR-140-3p, miR-532-3p, miR-194, miR-190, miR-204, miR-206	miR-206, miR-532-3	-
Sayilar, 2016 [40]	Turkey	30	15	RT-qPCR	plasma, urine	miR-21, miR-124, miR-192, miR-195, miR-451	Urine—miR-124 Plasma—miR-195, miR-451	Urine—miR-195, miR-451
Shang, 2017 [41]	China	208	37	RT-qPCR	serum	miR-92a, miR-126, miR-155, miR-483	miR-92a	-
Trojanowicz, 2019 [42]	Germany	48	23	RT-qPCR	serum	miR-421	miR-421	-
Ulbing, 2017 [43]	Austria	137	36	RT-qPCR	serum	miR-223-3p, miR-93-5p, miR-142-3p, miR-146a-5p	-	miR-223-3p, miR-93-5p, miR-142-3p

**Table 2:** Characteristics of studies evaluating microRNA expression patterns in diabetic kidney disease.

Study	Country	Study Population (n)			Quantification Method	microRNAs	Sample Type	Upregulated	Downregulated
		Healthy	Normoalbuminuria	Diabetic Kidney Disease					
Abdelsalam, 2020 [44]	Egypt	30	30	60	RT-qPCR	miR-451	plasma	miR-451	-
							urine	-	miR-451
Abdou, 2022 [45]	Egypt	20	20	40	RT-qPCR	miR-152-3p	serum	miR-152-3p	-
Akhbari, 2018 [46]	Iran	22	21	40	RT-qPCR	miR-93	serum	-	miR-93
Akhbari, 2019 [47]	Iran	22	-	61	RT-qPCR	miR-155	cell-free serum	-	miR-155
Al-kafaji, 2016 [48]	Bahrain	50	52	50	RT-qPCR	miR-126	peripheral whole blood	-	miR-126
Al-kafaji, 2018 [49]	Bahrain	30	30	25	RT-qPCR	miR-377, miR-192	whole blood	miR-377	miR-192
Argyropoulos, 2013 [50]	United States of America	-	10	30	RT-qPCR	27 microRNAs	urine	miR-214-3p, miR-92b-5p, miR-765, miR-429, miR-373-5p, miR-1913, miR-638	miR-323b-5p, miR-221-3p, miR-524-5p, miR-188-3p
Assmann, 2019 [51]	Brazil	20	33	54	RT-qPCR	miR-16-5p, miR-21-3p, miR-29a-3p, miR-378a-5p, miR-503-5p	plasma	miR-21-3p, miR-378a-5p	miR-16-5p, miR-29a-3p
Barutta, 2013 [52]	Italy	10	12	12	RT-qPCR	miR-130a, miR-424, miR-155, miR-145	urine exosomes	miR-145, miR-130a	miR-424, miR-155
Beltrami, 2018 [53]	United Kingdom	61	62	109	MicroRNA array, RT-qPCR	miR-126-3p, miR-155-5p, miR-29b-3p	urine	miR-126-3p, miR-155-5p, miR-29b-3p	-
Cardenas-Gonzalez, 2017 [54]	United States of America	93	71	132	RT-qPCR, miRNA in situ hybridization	miR-1915-3p, miR-2861, miR-4532, miR-4536-3p, miR-6747-3p	urine	miR-4536-3p, miR-6747-3p	miR-1915-3p, miR-2861, miR-4532
Conserva, 2019 [55]	Italy	20	-	37	Microarray, RT-qPCR	miR-27b-3p, miR-1228-3p	kidney biopsy, cell-free urine	-	miR-27b-3p, miR-1228-3p
Delić, 2016 [56]	Germany	14	14	13	Microarray, RT-qPCR	miR-320c, miR-6068	urine exosomes	miR-320c, miR-6068	-
Dieter, 2019 [57]	Brazil	-	17	23	RT-qPCR	miR-15a-5p, miR-30e-5p	plasma	-	miR-30e-5p
							urine	-	miR-30e-5p
Eissa, 2016 [58]	Egypt	56	60	116	MicroRNA array, RT-qPCR	miR-15b, miR-34a, miR-636	urine pellets, exosomes	miR-15b, miR-34a, miR-636	-
Eissa, 2016b [59]	Egypt	54	56	110	RT-qPCR	miR-133b, miR-342, miR-30a	urine exosomes	miR-133b, miR-342, miR-30a	-
Florijn, 2019 [60]	Netherlands	12	-	33	RT-qPCR	miR-1, miR-21, miR-29a, miR-126, miR-132, miR-145, miR-152, miR-212, miR-223, miR-574, miR-660	plasma endothelial vesicles	miR-21, miR-126	-
							Plasma	miR-126	
							high density lipoprotein fraction	-	miR-132
							Apo-2	miR-126, miR-145, miR-660	-
Fouad, 2020 [61]	Egypt	100	120	120	RT-qPCR	miR-21	plasma	miR-21	-
Guo, 2017 [62]	China	45	33	42	Microarray, RT-qPCR	miR-29c	plasma	miR-29c	-
							urine	-	miR-29c
							kidney tissue	-	miR-29c
Han, 2021 [63]	China	-	5	6	Microarray, RT-qPCR	miR-95-3p, miR-185-5p, miR-1246, miR-631	urine sediment	miR-95-3p, miR-185-5p, miR-1246, miR-631	-
He, 2014 [64]	China	6	-	6	Microarray hybridisation, RT-qPCR	miR-15a, miR-17, miR-21, miR-30b, miR-126, miR-135a, miR-192, miR-377, miR-34a, miR-194-1, miR-205, miR-215	serum	miR-15a, miR-17, miR-21, miR-30b, miR-126, miR-135a, miR-192, miR-377	miR-34a, miR-194-1, miR-205, miR-215
							kidney tissue	miR-135a	-
Hong, 2021 [65]	China	36	36	51	Microarray, RT-qPCR	miR-193a-3p, miR-320c, miR-27a-3p	plasma	miR-193a-3p, miR-320c	-
Jia, 2016 [66]	China	10	30	50	RT-qPCR	miR-192, miR-194, miR-215	urine extracellular vesicles	miR-192, miR-194, miR-215	-
Khokhar, 2021 [67]	India	36	38	35	RT-qPCR	miR-21-5p	whole blood	miR-21-5p	-
Lin, 2021 [68]	China	30	36	32	RT-qPCR	miR-638	serum		miR-638
Liu, 2021 [69]	China	180	64	116	RT-qPCR	miR-29a	serum	miR-29a	
Ma, 2016 [70]	China	127	157	307	RT-qPCR	miR-192	serum	-	miR-192
Milas, 2018 [71]	Romania	11	26	42	RT-qPCR	miR-21, miR-124, miR-192	urine	miR-21, miR-124	miR-192
Monjezi, 2022 [72]	Iran		30	31	RT-qPCR	miR-124-3p	peripheral blood mononuclear cells		miR-124-3p
Motawi, 2018 [73]	Egypt	25	25	25	RT-qPCR	miR-130b	serum	-	miR-130b
Park, 2022 [74]	Republic of Korea	7	-	12	NGS	miR-320b, miR-30d-5p, miR-30e-3p, miR-30c-5p, miR-190a-5p, miR-29c-5p, miR-98-3p, miR-331-3p, let-7a-3p, miR-106b-3p, miR-30b-5p, miR-99b-5p, let-7f-1-3p	plasma and urine extracellular vesicles	miR-320b	miR-30d-5p, miR-30e-3p, miR-30c-5p, miR-190a-5p, miR-29c-5p, miR-98-3p, miR-331-3p, let-7a-3p, miR-106b-3p, miR-30b-5p, miR-99b-5p, let-7f-1-3p
Peng, 2013 [75]	China	-	41	42	RT-qPCR	miR-29a, miR-29b, miR-29c	urine supernatant	miR-29a	-
Petrica, 2019 [76]	Romania	11	36	81	RT-qPCR	miR-125a, miR-126, miR-146a, miR-21p, miR-124, miR-192	serum	miR-192, miR-21p	miR-124, miR-125a, miR-126, miR-146
							urine	miR-21p, miR-124, miR-125a, miR-126	miR-192, miR-146a
Pezzolesi, 2015 [77]	United States of America	-	40	76	RT-qPCR	let-7b-5p, let-7c-5p, miR-21-5p, miR-29a-3p, miR-29c-3p	plasma	let-7b-5p, miR-21-5p	let-7c-5p, miR-29a-3p
Prabu, 2019 [78]	India	40	40	80	RT-qPCR	let-7i-5p, miR-135b-5p, miR-15b-3p, miR-197-3p, miR-24-3p, miR-27b-3p	urine exosomes	let-7i-5p, miR-24-3p, miR-27b-3p, miR-30a-5p	miR-15b-3p
Regmi, 2019 [79]	China	25	50	42	RT-qPCR	miR-20a, miR-99b, miR-122-5p, miR-486-5p	serum	miR-99b, miR-122	miR-20a, miR-486
Ren, 2019 [80]	China	280	273	465	RT-qPCR	miR-154-5p	serum	miR-154-5p	-
Ren, 2020 [81]	China	-	136	254	RT-qPCR	miR-154-5p	serum	miR-154-5p	-
Roux, 2018 [82]	France	-	73	73	NGS, RT-qPCR	miR-362-5p, miR-152-3p, miR-196b-5p, miR-140-3p	plasma	miR-152-3p	-
Rovira-Llopis, 2018 [83]	Spain	24	13	13	RT-qPCR	miR-31	serum	-	miR-31
Shao, 2017 [84]	China	195	186	309	RT-qPCR	miR-217	serum	miR-217	-
Sham, 2022 [85]	Malaysia	-	15	26	miS-cript miRNA qPCR array, RT-qPCR	miR-874-3p, miR-101-3p, miR-145-5p	serum	miR-874-3p, miR-101-3p	
Su, 2020 [86]	China	20	-	20	MicroRNA array, RT-qPCR	miR-140-5p	peripheral blood, kidney tissue	-	miR-140-5p
Wang, 2019 [19]	China	40	40	66	MicroRNA array, qPCR	miR-27a-3p, miR-30e, miR-33b, miR-50, miR-125b-5p, miR-150-5p, miR-155-5p, miR-296, miR-320e, miR-328, miR-484, miR-487, miR-550a-5p, miR-590-5p, miR-744, miR-885-5p, miR-933. miR-3196, let-7a-5p, let-7c-5p	plasma	miR-125b-5p, miR- 484, miR-550	miR-30e, miR-155-5p, miR-320, let-7a-5p, miR-150-5p, miR-3196
Xiao, 2017 [87]	China	35	-	140	Real time PCR	miR-9	serum	miR-9	-
Xie, 2017 [88]	China	-	35	5	MicroRNA array, qPCR	miR-362-3p, miR-877-3p, miR-15a-5p, miR-150-5p	urine exosomes	miR-362-3p, miR-877-3p, miR-150-5p	miR-15a-5p
Zang, 2019 [89]	Ireland	18	30	36	MicroRNA arrays, RT-PCR	miR-21-5p, let-7e-5p, miR-23b-3p, miR-30b-5p, miR-125b-5p	urine sediment exosome	miR-21-5p	miR-30b-5p
Zhang, 2017 [90]	China	28	30	27	Microarray, qPCR	miR-223-3p, miR-106b-5p, miR-103a-3p, miR-126-3p, miR-27a-3p, miR-29a-3p, miR-29c-3p, miR-425-5p, miR-93-5p, miR-1249-5p, miR-2276-3p, miR-1225-5p, miR-345-3p, miR-3679-5p, miR-4281, miR-4442	plasma	-	miR-223-3p
Zhang, 2020 [91]	China	-	30	30	RT-qPCR	miR-135a-5p	serum	miR-135a-5p	-
Zhao, 2020 [92]	China	-	17	17	MicroRNA arrays, qRT-PCR	miR-4491, miR-2117, miR-4507, miR-5088-5p, miR-1587, miR-219a-3p, miR-5091, miR-498, miR-4687-3p, miR-516b-5p, mir-4534, miR-1275, miR-5007-3p, miR-4516	urine exosomes	miR-4687-3p, miR-4534, miR-5007-3p	-
Zhou, 2013 [93]	China	62	104	108	MicroRNA microarrays, real time RT-PCR	let-7a, let-7d, let-7f, miR-4429, miR-363	whole blood	-	let-7a

**Table 3:** Characteristics of studies evaluating microRNA expression patterns in hypertension-associated chronic kidney disease.

Study	Country	Study Population			Quantification Method	Sample Type	microRNAs	Upregulated	Downregulated
		Healthy	Hypertensive	Hypertensive CKD					
Berillo, 2020 [94]	Canada	15	31	16	Hi-seq, RT-qPCR	platelet-poor plasma	let-7g-5p, miR-191-5p	-	let-7g-5p, miR-191-5p
Huang, 2018 [95]	China	0	50	100	RT-qPCR	plasma	miR-29a	miR-29a	-
Huang, 2020 [96]	China	0	50	100	RT-qPCR	plasma	miR-29b	miR-29b	-
Nandakumar, 2017 [97]	United States of America	-	15	15	NGS	whole blood	miR-17-5p, miR-130a-3p, miR-15b-5p, miR-106b-3p, miR-106a-5p, miR-16-5p, miR-181a-5p, miR-1285-3p, miR-15a-5p, miR-29c-5p, miR-345-5p, miR-142-3p, miR-339-3p, miR-210-3p	-	miR-17-5p, miR-15a-5p, miR-15b-5p, miR-16-5
Perez-Hernandez, 2018 [98]	Spain	20	28	24	NGS, RT-qPCR	Urinary exosomes	miR-146a and miR-335	-	miR-146a
Perez-Hernandez, 2021 [99]	Spain	15	56	61	NGS, RT-qPCR	plasma and urine exosomes	miR-143-3p, miR-126-3p, miR-26a-5p, miR-144-3p, miR-191-5p, miR-220a-3p, miR-222-3p, miR-423-5p	Plasma exosome—miR-191-5p	Plasma exosome—miR-222-3p, miR-26a-5p, miR-126-3p

**Table 4:** MicroRNAs reported in at least three studies in chronic kidney disease subtypes.

MicroRNA	Study	Sample Type	Expression Pattern
**CHRONIC KIDNEY DISEASE**			
miR-126	Carmona, 2020 [28]	Serum	Downregulated
	Fourdinier, 2019 [21]	Serum	Downregulated
	Fujii, 2019b [32]	Serum	Downregulated
	Shang, 2017 [41]	Serum, urine	No difference
miR-223	Carmona, 2020 [28]	Serum	Downregulated
	Fourdinier, 2019 [21]	Serum	Downregulated
	Fujii, 2019b [32]	Serum	Downregulated
	Ulbing, 2017 [43]	Serum	Downregulated
miR-155	Chen, 2013 [29]	Serum	Downregulated
	Donderski, 2021 [30]	Urine	Downregulated
		Serum	Upregulated
	Eckersten, 2017 [31]	Serum	Upregulated
	Shang, 2017 [41]	Serum, urine	No difference
miR-21	Donderski, 2021 [30]	Urine	Upregulated
		Serum	Downregulated
	Lange, 2019 [34]	Urine exosomes	Upregulated
	Sayilar, 2016 [40]	Urine, plasma	No difference
**DIABETIC KIDNEY DISEASE**			
miR-155	Akhbari, 2019 [47]	Cell-free serum	Downregulated
	Barutta, 2013 [49]	Urinary exosomes	Downregulated in microalbuminuria
	Beltrami, 2018 [53]	Urine	Upregulated
	Wang, 2019 [19]	Plasma	Downregulated
miR-126	Al-kafaji, 2016 [48]	Whole blood	Downregulated
	Beltrami, 2018 [53]	Urine	Upregulated
	Florijn, 2019 [60]	Plasma exosomal vesicles	Upregulated
		Plasma	Upregulated
		Plasma Ago	Upregulated
	Petrica, 2019 [76]	Urine	Upregulated
		Serum	Downregulated
	He, 2014 [64]	Serum	Upregulated
miR-192	Al-kafaji, 2018 [49]	Whole blood	Downregulated
	Jia, 2016 [66]	Urine extracellular vesicles	Upregulated in microalbuminuria and downregulated in macro albuminuria
	Ma, 2016 [70]	Serum	Downregulated
	Milas, 2018 [71]	Urine	Downregulated
	Petrica, 2019 [76]	Urine	Upregulated
		Serum	Upregulated
	He, 2014 [64]	Serum	Upregulated
miR-21	Assmann, 2019 [51]	Plasma	Upregulated in macroalbuminuria
	Florijn, 2019 [60]	Plasma exosomal vesicles	Upregulated
		Plasma	No difference
	Fouad, 2020 [61]	Plasma	Upregulated
	Khokhar, 2021 [67]	Whole blood	Upregulated
	Milas, 2018 [71]	Urine	Upregulated
	Petrica, 2019 [76]	Serum	Upregulated
		Urine	Upregulated
	Pezzolesi, 2015 [77]	Plasma	Upregulated in rapid progressors to ESKD
	Zang, 2019 [89]	Urinary exosomes	Upregulated
	He, 2014 [64]	Serum	Upregulated
miR-29b	Beltrami, 2018 [53]	Urine	Upregulated
	Peng, 2013 [75]	Urine supernatant	No difference
	Argyropoulos, 2013 [50]	Urine	Upregulated
miR-15a-5p	He, 2014 [64]	Serum	Upregulated
	Xie, 2017 [88]	Urinary exosomes	No difference
	Dieter, 2019 [57]	Urine and plasma	No difference
miR-29a	Assmann, 2019 [51]	Plasma	Downregulated in macroalbuminuria
	Peng, 2013 [75]	Urine supernatant	Upregulated
	Pezzolesi, 2015 [77]	Plasma	Downregulated in fast progressors to ESKD
	Liu, 2021 [69]	Serum	Upregulated
miR-29c	Guo, 2017 [62]	Plasma	Upregulated
		Urine sediments	Downregulated
		Kidney tissue	Downregulated
	Pezzolesi, 2015 [77]	Plasma	No difference
	Peng, 2013 [75]	Urine supernatant	No difference
miR-124	Milas, 2018 [71]	Urine	Upregulated
	Monjezi, 2022 [72]	Peripheral blood mononuclear cells	downregulated
	Petrica, 2019 [76]	Serum	Downregulated
		Urine	Upregulated
Let-7a	Park, 2022 [74]	Plasma	Downregulated
		Urinary extracellular vesicles	Downregulated
	Wang, 2019 [19]	Plasma	Downregulated
	Zhou, 2013 [93]	Whole blood	Downregulated
miR-30e	Dieter, 2019 [57]	Plasma	Downregulated
		Urine	Downregulated
	Park, 2022 [74]	Plasma	Downregulated
		Urinary extracellular vesicles	Downregulated
	Wang, 2019 [19]	Plasma	Downregulated
miR-30b	He, 2014 [64]	Serum	Upregulated
	Park, 2022 [74]	Plasma	Downregulated
		Urinary extracellular vesicles	Downregulated
	Zang, 2019 [89]	Urine sediment exosome	Downregulated

**Table 5:** Association of miRNAs with kidney disease outcome.

Study	microRNA	Adjustment	Effect Estimate [OR (95%CI)]	Outcome
Fujii, 2019 [22]	miR-17	sex, age, proteinuria, body mass index, systolic blood pressure, triglyceride, blood glucose, smoking status, alcohol consumption, exercise habit, and medication for non-communicable diseases	0.42 (0.24 to 0.75); *p* = 0.004	CKD
	miR-21		0.47 (0.26 to 0.85); *p* = 0.01	
	miR-150		0.49 (0.27 to 0.88); *p* = 0.02	
Fujii, 2019b [32]	miR-126	age, sex, blood glucose, body mass index, systolic blood pressure, smoking status, alcohol consumption, relocation frequency, degree of housing damage, current housing environment, and psychological condition	0.67 (0.45 to 0.98); *p* = 0.04	CKD
	miR-197		0.67 (0.46 to 0.99); *p* = 0.05	
	miR-223		0.53 (0.35 to 0.79); *p* = 0.002	
Fujii, 2021 [33]	miR-126	Sex, age, body mass index, blood glucose levels, systolic blood pressure, smoking status, alcohol intake, habitual exercise, proteinuria and baseline eGFR or blood urea nitrogen	3.85 (1.01 to 16.8); *p* = 0.05	CKD
Huang, 2018 [95]	miR-29a	age, sex, SBP, fasting blood-glucose, body mass index, glomerular filtration rate, triglyceride, C-reactive protein, and TGF-β1	1.11 (1.08 to 1.37); *p* = 0.002	Proteinuria
Huang, 2020 [96]	miR-29b	age, gender, SBP, fasting blood-glucose, body mass index, glomerular filtration rate, low density lipoprotein cholesterol, C-reactive protein and TGF-β1	0.55 (0.35 to 0.79); *p* < 0.001	Albuminuria
Motshwari, 2021 [37]	miR-novel-chr2_55842	age, gender, smoking status, drinking status, HTN, and DM status	1.65 (1.33 to 2.05); *p* < 0.0001	CKD
	miR-novel-chr7_76196		4.89 (2.48 to 9.64); *p* < 0.0001	
	miR-novel-chr5_67265		1.37 (1.17 to 1.60); *p* < 0.0001	
	miR-novel-chr13_13519		1.79 (1.40 to 2.28); *p* < 0.0001	
	miR-novel-chr1_36178		1.22 (1.10 to 1.37); *p* < 0.0001	
	miR-novel-chr15_18383		1.44 (1.09 to 1.89); *p* = 0.009	
Al-kafaji, 2016 [48]	miR-126	age, gender, BMI and blood pressure, fasting glucose, HbA1c, triglyceride, and LDL	0.51 (0.37 to 0.71); *p* = 0.002	DKD
			0.78 (0.70 to 0.95); *p* = 0.04	Microalbuminuria
			0.43 (0.30 to 0.70); *p* = 0.03	Macroalbuminuria
Al-kafaji, 2018 [49]	miR-377	age, sex, BMI, HbA1c, mean blood pressure, LDL, triglyceride and total cholesterol	1.12 (0.98 to 1.22); *p* = 0.018	DKD
Cardenas-Gonzalez, 2017 [54]	miR-4536-3p	Not reported	3.03 (1.95 to 4.72)	DKD
Pezzolesi, 2015 [77]	let-7b-5p	Sex, age, HbA1c, duration of type 1 diabetes	2.51 (1.42 to 4.43); *p* = 0.002	ESKD
	miR-21-5p		6.33 (1.75 to 22.92); *p* = 0.005	
	let-7c-5p		0.23 (0.10 to 0.52); *p* = 0.0004	
	miR-29a-3p		0.38 (0.20–0.74); *p* = 0.004	

**Table 6:** Association of miRNAs with kidney function and damage.

Study	microRNA	Adjustment	Unstandardized/Standardized β-Coefficient (95%CI)	Outcome
Chen, 2013 [29]	miR-125b	Not reported	Not reported	eGFR
	miR-145			
	miR-155			
Donderski, 2021 [30]	miR-155-5p	Not reported	0.32; *p* = 0.042	eGFR
Fourdinier, 2019 [21]	miR-223	age, body mass index, diabetes, urea, calcium, phosphate, parathyroid hormone, platelet count, cholesterol, and low-density lipoprotein	0.02 (0.01 to 0.03); *p* < 0.0001	eGFR
	miR-126	hypertension, body mass index, diabetes, urea, phosphate, parathyroid hormone, proteinuria, cholesterol, and low-density lipoprotein	0.00 (0.000 to 0.001); *p* = 0.002	eGFR
Fujii, 2019 [22]	miR-17	sex, age, proteinuria, body mass index, systolic blood pressure, triglyceride, blood glucose, smoking status, alcohol consumption, exercise habit, and medication for non-communicable diseases	0.121; *p* = 0.004	eGFR
	miR-21		0.134; *p* = 0.002	
	miR-150		0.123; *p* = 0.004	
Fujii, 2021 [33]	miR-126	age, sex, smoking habits, alcohol intake, habitual exercise, BMI, SBP, glucose levels, proteinuria, and baseline eGFR	−3.18; *p* = 0.04	eGFR
Motshwari, 2021 [37]	miR-novel-chr2_55842	age, gender, smoking status, drinking status, hypertension, and diabetes mellitus status	−2.70 (−4.82 to −0.57); *p* = 0.013	eGFR
	miR-novel-chr7_76196		−7.39 (−14.05 to −0.72); *p* = 0.030	
Shang, 2017 [41]	miR-92a	age, sex, smoking, diabetes mellitus, coronary artery disease, and hyperlipidaemia	−0.684; *p* < 0.001 −0.548; *p* < 0.001	eGFR
Berillo, 2020 [94]	let-7g-5p	age, urinary albumin creatinine ratio, carotid distensibility, neutrophil and lymphocyte fractions, neutrophil number and neutrophil-to-lymphocyte ratio	0.41; *p* < 0.001	eGFR
	miR-191-5p		0.30; *p* < 0.014	
Eissa, 2016 [58]	miR-15b	Not reported	0.452 (0.000 to 0.000); *p* < 0.001	UACR
	miR-34a		−0.914 (0.000 to 0.000); *p* < 0.03	
	miR-636		0.889 (0.000 to 0.000); *p* < 0.02	
Eissa, 2016b [59]	miR-133b	Not reported	0.4 (0.395 to 1.855); *p* < 0.01	eGFR
Ma, 2016 [70]	miR-192	Age, duration, body mass index, systolic and diastolic blood pressure, fasting blood glucose, postprandial blood glucose, HbA1C, fasting insulin, postprandial insulin, fasting C peptides, prandial C peptides, blood urea nitrogen, creatinine, low- and high-density lipoprotein cholesterol, triglycerides, cholesterol, TGF-β1, and fibronectin	Not reported	UACR
Milas, 2018 [71]	miR-21	lipid profile, HbA1c, and high-sensitive C-reactive protein	−0.007 (−0.011 to −0.003); *p* = 0.0001	eGFR
	miR-124		−0.007 (−0.011 to −0.003); *p* = 0.0001	
	miR-192		0.005 (0.002 to 0.008); *p* = 0.0001	
	miR-21		−0.0005 (−0.0007 to −0.0002); *p* = 0.0001	UACR
	miR-124		−0.0005 (−0.0007 to −0.0002); *p* = 0.0001	
Xiao, 2017 [87]	miR-9	pigment epithelium-derived factor, vascular endothelial growth factor, low-density lipoprotein cholesterol, total cholesterol, fibrinogen, HbA1c, insulin resistance	0.431; *p* = 0.023	UAER

## Data Availability

All data generated during this study is available upon reasonable request from the corresponding author.

## Supplementary Materials

The following supporting information can be downloaded at: https://www.mdpi.com/article/10.3390/ijms24021792/s1.
