# Supplementary material for: MicroRNAs Associated with Chronic Kidney Disease in the General Population and High-Risk Subgroups—A Systematic Review
Source: Int J Mol Sci. 2023 Jan 16;24(2):1792. doi: 10.3390/ijms24021792 (PMC9863068; doi:10.3390/ijms24021792)
Supplement: Supplementary file 1 [file ijms-24-01792-s001.zip › Supplementary Files.pdf]

Supplementary data

Supplementary Table S1 Medline (PubMed) search strategy (from inception to 30 September 2022)

**Search**	**Query**	**Number of hits**
#1	(Chronic kidney disease) OR (chronic kidney failure) OR (chronic renal disease) OR (chronic renal failure) OR (end-stage renal disease) OR (end-stage renal failure) OR (diabetic kidney disease) OR (diabetic nephropathy) OR (hypertensive nephrosclerosis) OR albuminuria OR proteinuria OR (HIV associated nephropathy) OR HIVAN OR (HIV-associated kidney disease) OR (HIV-associated renal disease)	277,388
#2	(Serum creatinine) OR (serum cystatin C) OR (estimated glomerular filtration rate) OR (urinary albumin excretion rate) OR (albumin-to-creatinine ratio) OR (urinary albumin)	95,064
#3	microRNAs OR miRNA OR miRNAs	133,454
#4	animal OR rat OR mouse OR (cell-line)	7,721,509
#5	cancer OR (acute kidney injury)	4,215,872
#6	#1 OR #2	333,540
#7	#6 AND #3	1,627
#8	#7 NOT #	683
#9	#8 NOT #5	577

Supplementary Table S2 Web of science search strategy (from inception to 30 September 2022)

**Search**	**Query**	**Number of hits**
#1	(Chronic kidney disease) OR (chronic kidney failure) OR (chronic renal	

	disease) OR (chronic renal failure) OR (end-stage renal disease) OR (end-stage renal failure) OR (diabetic kidney disease) OR (diabetic nephropathy) OR (hypertensive nephrosclerosis) OR albuminuria OR proteinuria OR (HIV associated nephropathy) OR HIVAN OR (HIV-associated kidney disease) OR (HIV-associated renal disease)	244,799
#2	(Serum creatinine) OR (serum cystatin C) OR (estimated glomerular filtration rate) OR (urinary albumin excretion) OR (albumin-to-creatinine ratio) OR (urinary albumin)	87,982
#3	microRNAs OR miRNA OR miRNAs	117,582
#4	animal OR rat OR mouse OR (cell-line)	4,402,334
#5	cancer OR (acute kidney injury)	3,752,456
#6	(ALL= ((Chronic kidney disease) OR (chronic kidney failure) OR (chronic renal disease) OR (chronic renal failure) OR (end-stage renal disease) OR (end-stage renal failure) OR (diabetic kidney disease) OR (diabetic nephropathy) OR (hypertensive nephrosclerosis) OR albuminuria OR proteinuria OR (HIV associated nephropathy) OR HIVAN OR (HIV-associated kidney disease) OR (HIV-associated renal disease)) OR ALL= ((Serum creatinine) OR (serum cystatin C) OR (estimated glomerular filtration rate) OR (urinary albumin excretion) OR (albumin-to-creatinine ratio) OR (urinary albumin))	296,928
#7	((ALL=((Chronic kidney disease) OR (chronic kidney failure) OR (chronic renal disease) OR (chronic renal failure) OR (end-stage renal disease) OR (end-stage renal failure) OR (diabetic kidney disease) OR (diabetic nephropathy) OR (hypertensive nephrosclerosis) OR albuminuria OR proteinuria OR (HIV associated nephropathy) OR HIVAN OR (HIV-associated kidney disease) OR (HIV-associated renal disease)) OR ALL=((Serum creatinine) OR (serum cystatin C) OR (estimated glomerular filtration rate) OR (urinary albumin excretion) OR (albumin-to-creatinine ratio) OR (urinary albumin)) AND ALL=(microRNAs OR miRNA OR miRNAs)	1,554
#8	(((ALL=((Chronic kidney disease) OR (chronic kidney failure) OR (chronic renal disease) OR (chronic renal failure) OR (end-stage renal disease) OR	913

	(end-stage renal failure) OR (diabetic kidney disease) OR (diabetic nephropathy) OR (hypertensive nephrosclerosis) OR albuminuria OR proteinuria OR (HIV associated nephropathy) OR HIVAN OR (HIV-associated kidney disease) OR (HIV-associated renal disease)) OR ALL=((Serum creatinine) OR (serum cystatin C) OR (estimated glomerular filtration rate) OR (urinary albumin excretion) OR (albumin-to-creatinine ratio) OR (urinary albumin)) AND ALL=(microRNAs OR miRNA OR miRNAs)) NOT ALL=(animal OR rat OR mouse OR (cell-line))	
#9	((((ALL=((Chronic kidney disease) OR (chronic kidney failure) OR (chronic renal disease) OR (chronic renal failure) OR (end-stage renal disease) OR (end-stage renal failure) OR (diabetic kidney disease) OR (diabetic nephropathy) OR (hypertensive nephrosclerosis) OR albuminuria OR proteinuria OR (HIV associated nephropathy) OR HIVAN OR (HIV-associated kidney disease) OR (HIV-associated renal disease)) OR ALL=((Serum creatinine) OR (serum cystatin C) OR (estimated glomerular filtration rate) OR (urinary albumin excretion rate)) OR (albumin-to-creatinine ratio) OR (urinary albumin)) AND ALL=(microRNAs OR miRNA OR miRNAs)) NOT ALL=(animal OR rat OR mouse OR (cell-line))) NOT ALL=(cancer OR (acute kidney injury))	669

Supplementary Table S3 Scopus search strategy (from inception to 30 September 2022)

**Search**	**Query**	**Number of hits**
#1	"Chronic kidney disease" OR "chronic kidney failure" OR "chronic renal disease" OR "chronic renal failure" OR "end-stage renal disease" OR "end-stage renal failure" OR "diabetic kidney disease" OR "diabetic nephropathy" OR "hypertensive nephrosclerosis" OR albuminuria OR proteinuria OR “HIV associated nephropathy” OR HIVAN OR “HIV-associated kidney disease” OR “HIV-associated renal disease”	626,284
#2	"Serum creatinine" OR "serum cystatin C" OR "estimated glomerular filtration rate" OR " urinary albumin excretion rate" OR "albumin-to-creatinine ratio" OR "urinary albumin"	139,458
#3	microRNAs OR miRNA OR miRNAs	309,549
#4	animal OR rat OR mouse OR "cell-line"	

		13,190,253
#5	cancer OR "acute kidney injury"	7,436,396
#6	("Chronic kidney disease" OR "chronic kidney failure" OR "chronic renal disease" OR "chronic renal failure" OR "end-stage renal disease" OR "end-stage renal failure" OR "diabetic kidney disease" OR "diabetic nephropathy" OR "hypertensive nephrosclerosis" OR albuminuria OR proteinuria OR "HIV associated nephropathy" OR HIVAN OR "HIV-associated kidney disease" OR "HIV-associated renal disease") OR ("Serum creatinine" OR "serum cystatin C" OR "estimated glomerular filtration rate" OR " urinary albumin excretion rate" OR "albumin-to-creatinine ratio" OR "urinary albumin")	682,499
#7	(("Chronic kidney disease" OR "chronic kidney failure" OR "chronic renal disease" OR "chronic renal failure" OR "end-stage renal disease" OR "end-stage renal failure" OR "diabetic kidney disease" OR "diabetic nephropathy" OR "hypertensive nephrosclerosis" OR albuminuria OR proteinuria OR "HIV associated nephropathy" OR HIVAN OR "HIV-associated kidney disease" OR "HIV-associated renal disease") OR ("Serum creatinine" OR "serum cystatin C" OR "estimated glomerular filtration rate" OR "urinary albumin excretion rate" OR "albumin-to-creatinine ratio" OR "urinary albumin")) AND (microRNAs OR miRNA OR miRNAs)	13,330
#8	((("Chronic kidney disease" OR "chronic kidney failure" OR "chronic renal disease" OR "chronic renal failure" OR "end-stage renal disease" OR "end-stage renal failure" OR "diabetic kidney disease" OR "diabetic nephropathy" OR "hypertensive nephrosclerosis" OR albuminuria OR proteinuria OR "HIV associated nephropathy" OR HIVAN OR "HIV-associated kidney disease" OR "HIV-associated renal disease") OR ("Serum creatinine" OR "serum cystatin C" OR "estimated glomerular filtration rate" OR " urinary albumin excretion rate" OR "albumin-to-creatinine ratio" OR "urinary albumin")) AND (microRNAs OR miRNA OR miRNAs)) AND NOT (animal OR rat OR mouse OR "cell-line")	1,505
#9	(((("Chronic kidney disease" OR "chronic kidney failure" OR "chronic renal disease" OR "chronic renal failure" OR "end-stage renal disease" OR "end-stage renal failure" OR "diabetic kidney	568

	disease" OR "diabetic nephropathy" OR "hypertensive nephrosclerosis" OR albuminuria OR proteinuria OR “HIV associated nephropathy” OR HIVAN OR “HIV-associated kidney disease” OR “HIV-associated renal disease”) OR ("Serum creatinine" OR "serum cystatin C" OR "estimated glomerular filtration rate" OR " urinary albumin excretion rate" OR "albumin-to-creatinine ratio" OR "urinary albumin")) AND (microRNAs OR miRNA OR miRNAs)) AND NOT (animal OR rat OR mouse OR "cell-line")) AND NOT (cancer OR "acute kidney injury")	

Supplementary Table S4 EBSCOhost search strategy (from inception to 30 September 2022)

Search	Query	Number of hits
#1	(Chronic kidney disease) OR (chronic kidney failure) OR (chronic renal disease) OR (chronic renal failure) OR (end-stage renal disease) OR (end-stage renal failure) OR (diabetic kidney disease) OR (diabetic nephropathy) OR (hypertensive nephrosclerosis) OR albuminuria OR proteinuria OR (HIV associated nephropathy) OR HIVAN OR (HIV-associated kidney disease) OR (HIV-associated renal disease)	430,313
#2	(Serum creatinine) OR (serum cystatin C) OR (estimated glomerular filtration rate) OR (urinary albumin excretion rate) OR (albumin-to-creatinine ratio) OR (urinary albumin)	127,450
#3	microRNAs OR miRNA OR miRNAs	219,791
#4	animal OR rat OR mouse OR (cell-line)	11,974,642
#5	cancer OR (acute kidney injury)	5,633,922
#6	#1 OR #2	506,221
#7	#6 AND #3	2,460
#8	#7 NOT #4	1,205
#9	#8 NOT #5	

		839
